# miR-766-3p Targeting BCL9L Suppressed Tumorigenesis, Epithelial-Mesenchymal Transition, and Metastasis Through the β-Catenin Signaling Pathway in Osteosarcoma Cells

**DOI:** 10.3389/fcell.2020.594135

**Published:** 2020-10-07

**Authors:** Sheng Zhang, Hongtao Chen, Wanshun Liu, Le Fang, Zhanyang Qian, Renyi Kong, Qi Zhang, Juming Li, Xiaojian Cao

**Affiliations:** ^1^Department of Orthopedics, First Affiliated Hospital of Nanjing Medical University, Nanjing, China; ^2^Department of Critical Care Medicine, First Affiliated Hospital of Nanjing Medical University, Nanjing, China; ^3^Department of Orthopedics, The Affiliated Zhongda Hospital of Southeast University, Nanjing, China; ^4^Department of Painology, Sir Run Run Hospital, Nanjing Medical University, Nanjing, China

**Keywords:** osteosarcoma, miR-766-3p, BCL9L, β-catenin, EMT, metastasis

## Abstract

Accumulating evidence has indicated that abnormal microRNAs (miRNAs) serve critical roles in carcinogenesis and development of osteosarcoma (OS). The purpose of the present study was to elucidate the relationship between miR-766-3p and development of osteosarcoma and explore the potential mechanism. In this study, we found that miR-766-3p was the most downregulated miRNA by analyzing GSE65071 from the GEO database. miR-766-3p was lowly expressed in OS tissue samples and cells, and high miR-766-3p expression repressed the malignant level of OS, including cell proliferation, EMT, migration, and invasion *in vitro* and *in vivo*. B-Cell Lymphoma 9-Like Protein (BCL9L) was negatively associated with miR-766-3p expression in OS cells and tissue samples and was validated as the downstream target by luciferase reporter assay and western blotting. Rescue experiment indicated that BCL9L could restore the influence of miR-766-3p on OS cells. In addition, the β-Catenin/TCF-4 signal pathway was demonstrated to be related to the miR-766-3p/BCL9L axis. In summary, miR-766-3p, a negative regulator of BCL9L, plays the role of tumor metastasis suppressor via the β-catenin signaling pathway in the progression of OS.

## Introduction

Osteosarcoma (OS) is a kind of primary high-grade malignant bone neoplasm originating from mesenchymal cells and has a high mortality rate all over the world (Ottaviani and Jaffe, 2009). Despite the continuous development of treatment methods such as chemotherapy and various surgical methods recently, the 5-year survival rate of osteosarcoma is still <70% (Kansara et al., 2014; Isakoff et al., 2015). It has a high potential for distant metastasis, particularly lung metastasis, and the 5-year overall survival rate is <30% (Aljubran et al., 2009; Luetke et al., 2014). Therefore, in order to develop new potent therapeutic strategies, the underlying mechanism of OS metastasis must be elucidated.

During the initiation of metastasis process, epithelial–mesenchymal transition (EMT) plays a vital role. EMT is a reversible phenotypic change in which polar epithelial cells acquire characteristics of mesenchymal cells and lose their epithelial properties, which may help explain tumorigenesis and metastasis of OS (Kudo-Saito et al., 2009; Craene and Berx, 2013; Brabletz et al., 2018). Therefore, inhibiting the progression of EMT may be a potentially therapeutic approach to OS treatment. MicroRNA (miRNA) is an endogenous small non-coding RNA, 22–28 nucleotides in length, which inhibits target gene by binding to the 3′-untranslated regions (3′-UTR) of the mRNA and degrading or translational inhibiting them (Berindan-Neagoe et al., 2014; Rupaimoole and Slack, 2017). Accumulating evidence demonstrates that miRNAs act as the vital role in the occurrence and progress of various tumors by diverse signal pathways (Rupaimoole et al., 2016). miRNAs are also identified important in the progression of OS by regulating cell proliferation, EMT, and metastasis (Leichter et al., 2017; Fellenberg et al., 2019; Luo et al., 2019; Zhang K. et al., 2020). miR-766-3p, a tumor suppressor microRNA, is reported to down-regulated in several types of tumor, was remarkably correlated with poor clinical outcomes (Liu et al., 2020; Zhou et al., 2020). Nevertheless, to date, there has been no detailed investigations into the functions of miR-766-3p, and the underling mechanisms is far from being fully understood in OS.

miR-766-3p detected in hepatocellular carcinoma (HCC) cells and tissues was lower than control samples, and was remarkedly associated with TNM stage, tumor size, metastasis, and poor prognosis of hepatocellular carcinoma by targeting Wnt family member 3A, metastasis-associated protein 3 and fos-related antigen 2 (You et al., 2018; Liu et al., 2019; Li et al., 2020). Furthermore, miR-766–3p suppressed the cell-cycle progression of renal cancer by regulating SF2 amplification and downstream signal pathway, which was markedly correlated with a prognosis of renal cell carcinoma (Chen et al., 2017). However, as we know, the precise role of miR-766-3p in osteosarcoma occurrence and metastasis is still unclear.

B-Cell Lymphoma 9-Like Protein (BCL9L), as a cofactor for canonical Wnt signaling, is a component of BCL9 family and mediates EMT (Gay et al., 2019). It has been reported that BCL9L induces early phases of human intestinal cancer progress by regulating the β-catenin function switch between adhesion and transcription (Brembeck et al., 2004; Huge et al., 2020). Moreover, BCL9L is demonstrated to enhance β-catenin–mediated transcriptions and increase the abilities of proliferation and metastasis in breast and colon cancer cell lines (Brembeck et al., 2011; El-Hage et al., 2015). However, the effects of BCL9L in osteosarcoma has never been clearly reported, and the relationship between the miR-766-3p/BCL9L axis and β-catenin signal pathway involved in OS is still subject to intense investigation.

In the study, we demonstrated that miR-766-3p was markedly down-regulated in osteosarcoma cells and tissue samples. And overexpressing miR-766-3p suppressed proliferation, EMT and metastasis in osteosarcoma by downregulating the expression of BCL9L via the β-catenin/TCF-4 signaling pathway.

## Materials and Methods

### OS Tissue Samples

The Ethics Committee of the First Affiliated Hospital of Nanjing Medical University approved our study. All experiments were conducted according to the approved guideline and regulation, and all subjects signed the written informed consent. 60 pairs of osteosarcomas and adjacent tissues were obtained from the patients undergoing biopsy before chemotherapy at the orthopedics department. Biopsy samples were collected and subsequently frozen by liquid nitrogen. All patients’ demographic and clinical information in this study is shown in Table 1.

**TABLE 1:** Expression of miR-766-3p and BCL9L according to patients’ clinical features.

		**miR-766-3p expression**			**BCL9L expression**		
**Characteristics**	**Total**	**High group**	**Low group**	***P*-value**	**High group**	**Low group**	***P*-value**
**Age (y)**							
<18	35	17	18	0.73	18	17	0.97
≥18	25	11	14		13	12	
**Gender**							
Female	28	15	13	0.45	14	15	0.61
Male	32	14	18		17	14	
**Location**							
Femur/Tibia	45	22	23	0.55	21	20	0.63
Elsewhere	15	6	9		11	8	
**TNM stage**							
I	27	14	13	0.02*	9	18	0.01*
II/III	33	8	25		22	11	
**Tumor size (cm)**							
< 5	31	17	14	0.032*	11	19	0.01*
≥ 5	29	8	21		21	9	
**Lung metastasis**							
Yes	26	4	22	0.005*	17	7	0.008*
No	34	17	17		13	23	

*P < 0.05 (Chi-square test).

### Cells and Cell Culture

All OS cells, including Saos-2, MG63, HOS, 143B and U2OS, and hFOB1.19 were purchased from the ATCC (American Type Culture Collection) (Manassas, United States). OS cells were cultured in DMEM/F12 medium (Life Technologies, NY) with 10% fetal bovine serum (Gibco, NY) and streptomycin/penicillin (1%, Gibco, CA) and incubated with 5% CO_2_ at 37°C until 80–85% confluence. The hFOB 1.19 cells were cultured in DMEM/F-12 (Life Technologies, NY) containing 10% fetal bovine serum (Gibco, NY), streptomycin/penicillin (1%, Gibco, CA) and 0.3 mg/mL of G418 and incubated with 5% CO_2_ at 33.5°C.

### Lentivirus Construction Establishment and Transfection

Synthesis of lentiviruses vector of pLV-hsa-miR-766-3p-pre-microRNA (miR-766-3p mimics), inhibitor vector of pLV-hsa-miR-766-3p-sponge (miR-766-3p inhibitor), vector including BCL9L sequence (BCL9L) was authorized to GenePharma (Shanghai, China). Osteosarcoma cells transfection was conducted in accordance with the manufacturer’s protocol of Lipofectamine 2000 (Invitrogen, CA, United States).

### qRT-PCR

The total RNAs from cells or tissue samples in Trizol (Invitrogen, United States) were extracted from the pulverized samples stored at liquid nitrogen. And the concentration and purity of total RNA were determined using the absorbance at 260 nm and 280 nm with UV-spectrophotometry (NanoDrop-2000, Massachusetts, United States). The primers of BCL9L, U6, miR-766-3p, and GAPDH were obtained from TsingKe (Beijing, China). RT (Reverse transcription) was undertaken with the GoldenstarTM RT6 cDNA Synthesis Kit (TsingKe, China) according to the protocol of manufacturer, and qPCR was conducted by SYBR Green Master (TsingKe, China). U6 or GAPDH expression functioned as the endogenous control. The primer sequences are shown in Supplementary Table S1.

### Western Blotting

After protein extraction, the concentration of protein was conformed with BCA Protein Quantification Kit (Thermo Fisher Scientific, United States). And Proteins were subjected to electrophoresis and then polyvinylidene difluoride membranes (Bio-Rad, United States). Next, proteins were incubated with specific primary antibodies, washed with PBST followed by incubated with the secondary antibody (goat-anti-Rabbit IgG, 1:10,000, Proteintech). And we used a panel of antibodies to detect these proteins, including Rabbit anti-BCL9L (1:1,000, Abcam), N-cadherin (1:1,000, Abcam), GAPDH (1:10,000, Proteintech), E-cadherin (1:1,000, Abcam), β-catenin (1:500, Abcam), Histone (1:5,000, Proteintech), Vimentin (1:1,000, Abcam), TCF-4 (1:1,000, Abcam), Cyclin D1 (1:1,000, Abcam) and Axin2 (1:1,000, Abcam) antibodies. Reacting bands were captured using the enhanced chemiluminescence (ECL) reagent (Tanon Technology Co., Ltd., China) and quantitative analysis was normalized to GAPDH or Histone by ImageJ software.

### Migration Assays

Cell migration ability was confirmed by transwell migration and wound-healing assays. Transwell chambers (8 μm pore size; Costar, NY, United States) were applied in the migration assay. In Brief, the Transwell co-culture assay was performed using the 12-well Transwell plate (Corning, MA, United States). Total of 1 × 10^4^ cells were incubated in the upper chamber with 200 μL of serum-free DMEM/F12, and 800 μL of DMEM/F12 with 10% FBS was added to the lower chamber. After 24 h incubation, cells, passing through the membrane, were fixed with 4% paraformaldehyde and stained by 0.1% crystal violet for 15 min. Finally, cells on the membrane were visualized and five random fields of each well were photographed with a microscope (Nikon, Japan), and cell numbers of five random fields were counted. For wound-healing experiment, cells grew to 90% confluence in six-well plates and were scratched by sterile 200 μL pipette tips. The wound closure was observed and was imaged under a microscope at 0 and 24 h.

### Invasion Assay

To assess the cell invasive ability, transwell invasion assays were performed. In Matrigel invasion assay, cells were inoculated on the upper chambers coated with 80 μL of Matrigel matrix (Millipore, United States) and the remaining steps were the same as with the migration assay. Following incubation for 24 h, the invasive cells were fixed, stained, counted and photographed under a light microscope.

### Colony Formation and CCK-8 Assays

The OS cells were seeded into six-well plates (500 cells per well) and cultured in DMEM containing 10% FBS for 2 weeks. When the colonies were visible to naked eyes, they were fixed with 4% paraformaldehyde and stained using 0.1% crystal violet. the images of all colonies were taken with a scanner (Microtek, China) and the colonies were counted manually. As for CCK-8 assay, the transfected OS cells were plated and cultured in 96-well plates. After incubating for 1, 2, 3, 4, or 5 days, cell proliferative rates were examined with the CCK-8 kit (Dojindo, Japan) following the instruction of manufacturer.

### 5-Ethynyl-2-Deoxyuridine (EdU) Incorporation Assays

The EdU incorporation assays were conducted in accordance with the manufacturer’s protocol of a kFluor 555 Click-iT EdU Imaging Kit (KeyGEN, Nanjing, China). At least 50 cells were randomly selected from each field. For fluorescence intensity quantification, the intensity was calculated from five different fields. And the images were captured by fluorescent microscope (Carl Zeiss, Germany).

### Immunofluorescence Analysis

Transfected cells were fixed with 4% paraformaldehyde for 10 min, permeabilized in 0.3% Triton X-100 for 15 min. Subsequently, these cells were immunofluorescence stained with the primary antibodies to β-catenin (1:250, Abcam, United States) overnight at 4°C. Fluorescein-conjugated secondary antibody and DAPI were then used to stain the nucleus and mount the sections. Ultimately, Images were collected by fluorescence microscope (Carl Zeiss, Germany).

### Luciferase Reporter Assay

We obtained possible miRNA-766-3p-binding sites from the TargetScan^1^ database. The wild-type and mutant 3′-UTR of BCL9L were synthesized by GenePharma (Shanghai, China). The cells upregulating miR-766-3p or their controls were compared with cells transfected with wild-type and mutant 3′-UTR of BCL9L. The cells were obtained after 48 h of transfection. Luciferase activity was detected by double Luciferase Assay System (Promega, United States), and normalized by the co-expressed Renilla luciferase.

### Immunohistochemical Staining

Immunohistochemical staining was conducted according to our previous study (Qian et al., 2020). Tissue samples were fixed, embedded, and cut into 5-μm sections. Then, the sections were incubated with the primary antibody of BCL9L (Abcam, United States), the secondary antibody and stained using the developed diaminobenzidine (DAB). The staining result was measured by combining the percentage of positive staining and intensity of positively stained tumor cells.

### Xenograft Transplantation Experiments

The Animal Ethics Committee of Nanjing Medical University approved animal studies in this study. Female BALB/c nude mice were purchased from Animal Core Facility of Nanjing Medical University (Nanjing, China) and were randomly assigned into four groups, five in each group. The OS cells with firefly luciferase were seeded into these nude mice. Tumor growth was observed every 3 days. The size of tumors was expressed using tumor volume and calculated with the formula: V = A × B2/2 (mm^3^), in which “A” referred to a larger diameter and “B” was a smaller diameter. On day 35 post injection, the imaging system of IVIS200 (Caliper Life Sciences, United States) was used to image the progression of xenograft growth.

### Statistical Analysis

All data from at least three times assays were presented as means ± standard deviation (SD). The association between miR-766-3p expression and clinicopathological characteristics was analyzed by χ^2^ test. The differences between the two group were compared using Independent Student’s *t*-test. Statistical analysis of significant differences in mRNA expression of BCL9L and miR-766-3p in paired tissues were conducted by paired *t*-test. Multivariate analysis of one-way or two-way ANOVA was using Bonferroni *post-hoc* test. Bivariate correlation was using Pearson’s correlation analysis. All statistical analyses were performed with SPSS, v. 20.0 (SPSS Inc., United States). The statistical significance *P* < 0.05 was considered statistically significant.

## Results

### miR-766-3p Expression Was Down-Regulated in OS Cells and Tissue Samples

**FIGURE 1:**
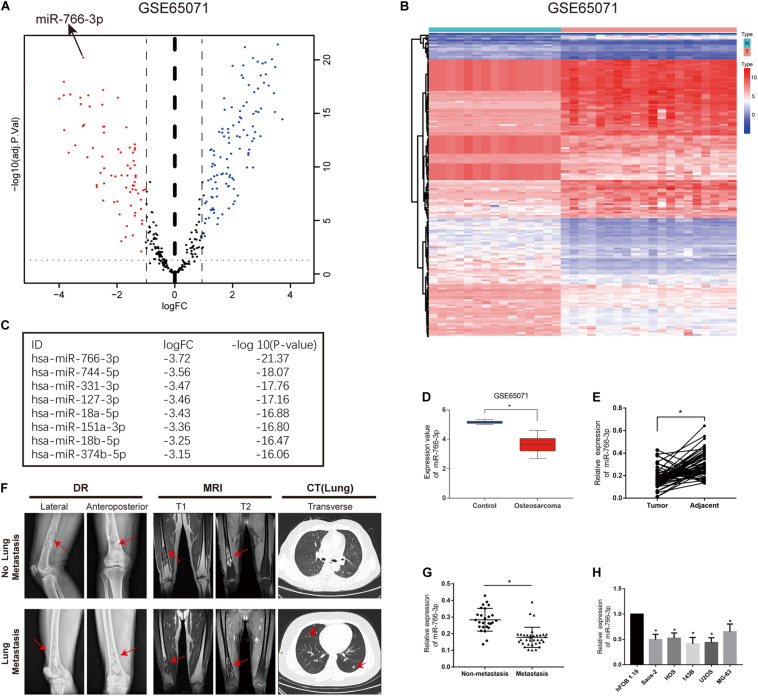
miR-766-3p is downregulated in osteosarcoma (OS) cell lines and clinical tissues. **(A)** Volcano plot compared the differentially expressed miRNAs between OS and normal tissues from GSE65071. **(B)** The cluster heap map showed the up-regulated and down-regulated microRNAs (miRNAs) in GSE65071. **(C)** The top eight downregulated miRNAs are listed. **(D,E)** Expression of miR-766-3p was markedly downregulated in OS clinical tissues from GSE65071 and in-house cohort. **(F)** Representative X-ray, computed tomography (CT) and Magnetic resonance imaging (MRI) images of OS patients with and without pulmonary metastasis. **(G)** miR-766-3p was notably downregulated in patients according to in-house cohort with metastasis. **(H)** The relative expression of miR-766-3p was significantly decreased in OS cell lines (*n* = 4). Data are presented as the means ± SD. *P < 0.01.

In order to detect miRNAs expression in OS clinical tissues, the data from the GEO database (GSE65071) were analyzed using the R package limma (Wendy Allen-Rhoades et al., 2015). The volcano plot demonstrated the miRNA expression differences in normal and OS tissues (Figure 1A). According to the levels of miRNA expression, the up- and downregulated microRNAs was represented in a cluster heap map (Figure 1B). Totally, 70 miRNAs were downregulated (fold change > 2.0, FDR < 0.05) in OS tissues and the top eight most significantly differentially expressed miRNAs are listed in Figure 1C. We found the most down-regulated miRNA was miR-766-3p in these miRNAs. So, the molecular mechanism of the inhibiting influence of miR-766-3p became the target of our research. As demonstrated in Figure 1D, miR-766-3p was remarkedly low-expressed in OS tissues according to the data from GSE65071. Then, qPCR was performed to measure miR-766-3p expression in 60 paired tumor and peritumor tissues, and the results indicated that miR-766-3p was markedly down-regulated in tumor tissues (Figure 1E, *P* < 0.01). As shown in representative imaging pictures, the red arrows indicated tumor foci or pulmonary metastatic nodules (Figure 1F). And down-regulating miR-766-3p was found in patients with metastasis (Figure 1G, *P* < 0.01). In addition, compared with in hFOB 1.19 cells, the expression of miR-766-3p significantly decreased in OS cells (Figure 1H, *P* < 0.05). In order to identify the clinical significance of miR-766-3p, median level was defined as the critical value and the patients were divided into subgroups. The miR-766-3p expression was markedly negatively in correlation with tumor size, TNM stage, and lung metastasis (Table 1).

### Down-Regulation of miR-766-3p Promoted OS Cell EMT, Migration, and Invasion *in vitro*

**FIGURE 2:**
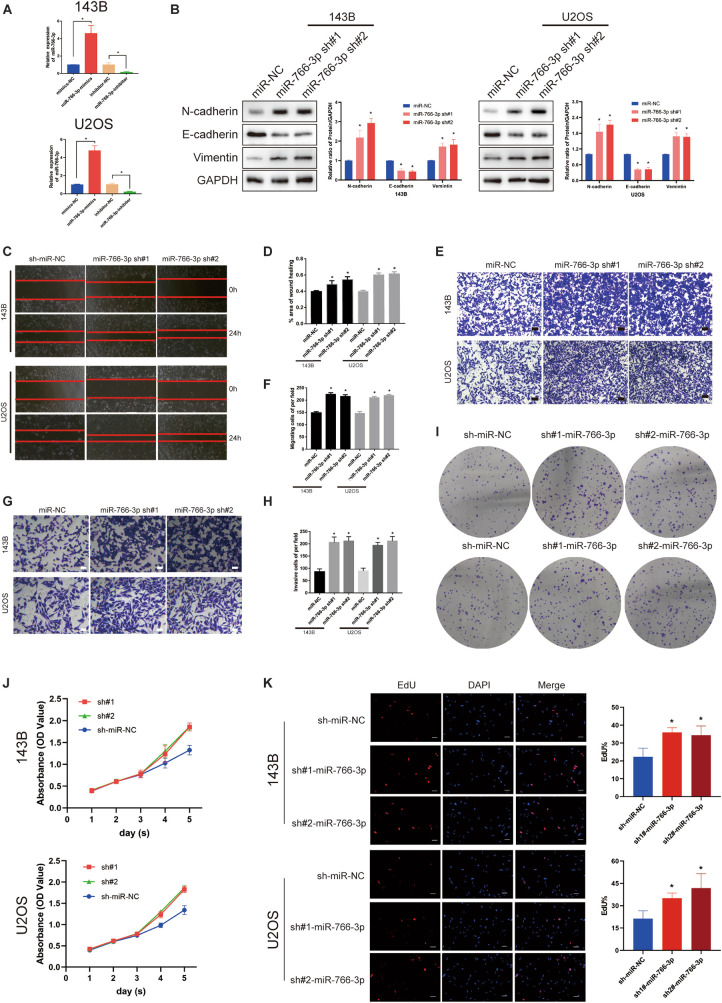
Downregulating miR-766-3p promoted OS cell EMT, migration and invasion *in vitro*. **(A)** miR-766-3p lentiviruses were successfully transfected into 143B and U2OS cell lines (*n* = 3). **(B)** miR-766-3p sh#1 and miR-766-3p sh#2 increased the expression level of metastasis-related proteins in 143B and U2OS (*n* = 3). **(C–F)** The knockdown of miR-766-3p notably promoted the invasion and migration of 143B and U2OS cells (*n* = 4). **(G,H)** The Transwell invasion assays indicated that the knockdown of miR-766-3p significantly increased the invasive ability of OS cells (*n* = 4). **(I–K)** Colony formation, CCK-8 and EdU assays demonstrated that downregulating miR-766-3p promoted the proliferation of OS cells (*n* = 4). Data are presented as the means ± SD. *P < 0.01.

Among these cell lines, 143B and U2OS cells were used to study further experiments *in vitro*. The transfection efficiency of miR-766-3p was verified lentiviruses using qRT-PCR (*P* < 0.001; Figure 2A). Western blotting experiment demonstrated that sh-miR-766-3p diminished the level of E-cadherin, but raised N-cadherin and vimentin expression in U2OS and 143B cell lines (Figure 2B). To explicate the influence of miR-766-3p on the ability of OS cell migration *in vitro*, transwell migration and wound-healing assays were conducted and the results demonstrated that down-regulated expression of miR-766-3p could promote OS cells migration (*P* < 0.01; Figures 2C–F). The transwell invasion assay demonstrated that down-regulation of miR-766-3p remarkedly increased invasiveness of OS cell lines (*P* < 0.01; Figures 2G,H). And the effects of miR-766-3p on cell proliferation were detected by CCK-8, EDU, and colony formation experiments. As can be seen from Figures 2I–K, downregulating miR-766-3p markedly promoted the proliferation of OS cells. Taken together, miR-766-3p has the potential to be an inhibitor in proliferation, EMT, and metastasis of OS cells.

### Overexpressing miR-766-3p Inhibited OS Cell EMT, Migration, and Invasion *in vitro*

**FIGURE 3:**
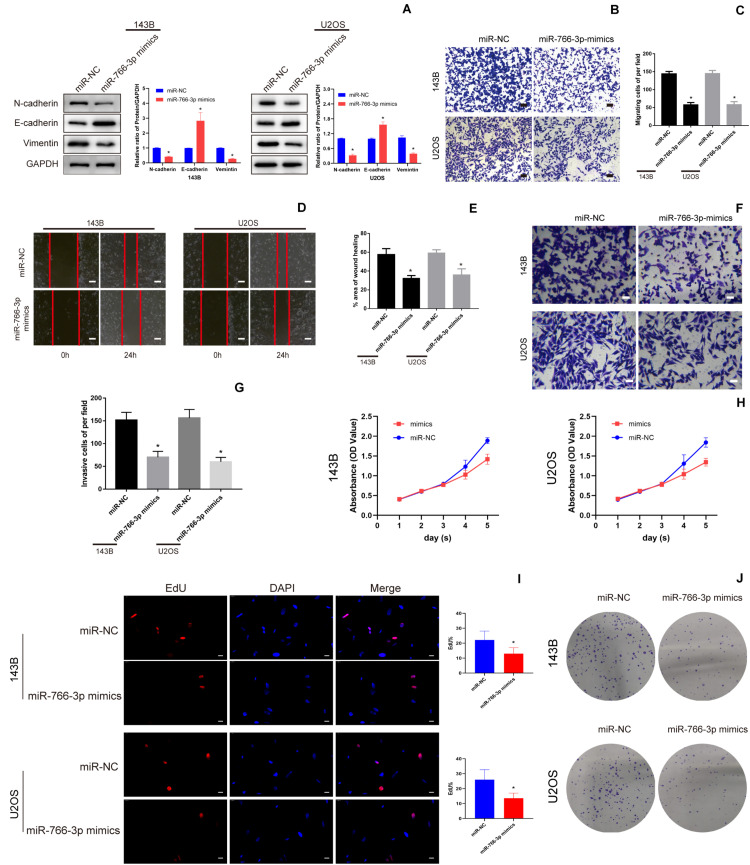
miR-766-3p suppressed OS cell EMT, migration, and invasion *in vitro*. **(A)** Western blotting demonstrated that miR-766-3p inhibited the EMT progression (*n* = 3). **(B–E)** Upregulating miR-766-3p remarkably suppressed cell migration in 143B and U2OS cells (*n* = 4). **(F–G)** Overexpression of miR-766-3p inhibited cell invasion in 143B and U2OS lines (*n* = 4). **(H–J)** Colony formation, CCK-8 and EDU assays showed that overexpressing miR-766-3p inhibited the proliferation of OS cells (*n* = 4). Data are presented as the means ± SD. *P < 0.01.

Western blotting revealed that miR-766-3p overexpression increased the E-cadherin level and deceased the levels of the Vimentin and N-cadherin which were related with metastasis in OS cell lines (Figure 3A). And transwell migration assays indicated that upregulating miR-766-3p remarkably decreased the ability of OS cells migration (*P* < 0.01; Figures 3B,C), and the wound-healing assay supported the above results (*P* < 0.01; Figures 3D,E). Then, to investigate the influence of miR-766-3p on invasion, Matrigel invasion assay was conducted and the result was consistent with the wound-healing and transwell migration assays (*P* < 0.01; Figures 3F,G). Moreover, CCK-8, colony formation, and EDU assays indicated that miR-766-3p markedly decreased OS cell proliferation (Figures 3H–J). It was indicated that miR-766-3p mediated the OS cells proliferation, EMT, migration, and invasion.

### BCL9L Was a Target Gene of miR-766-3p in OS Cell Lines and Tissues

**FIGURE 4:**
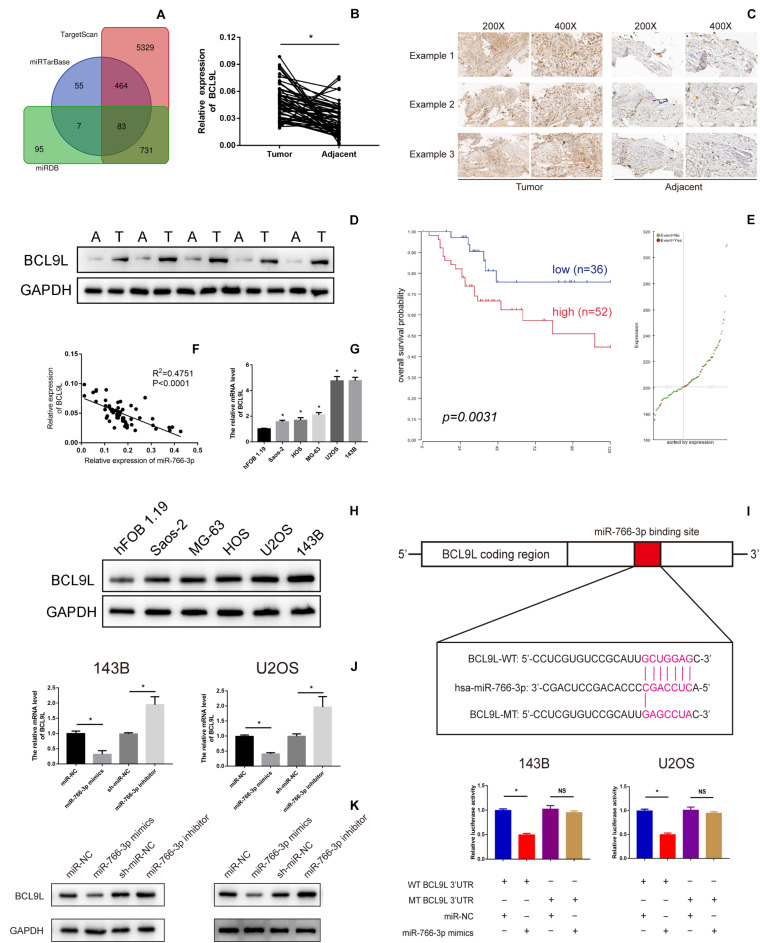
B-Cell Lymphoma 9-Like Protein (BCL9L) expression was upregulated in OS cell lines and tissues and was a target of miR-766-3p. **(A)** Bioinformatics analysis showed that hsa-miR-766-3p has a total of 83 target genes in miRDB, miRTarBase, and TargetScan. **(B–D)** BCL9L expression was remarkedly higher in OS tissues. **(E)** *Kaplan-Meier* analysis demonstrated that patients with low BCL9L expression levels had a better prognosis according to an online database (https://hgserver1.amc.nl/cgi-bin/r2/main.cgi). **(F)** Expression level of BCL9L was negatively correlated with miR-766-3p in OS tissues. **(G)** The mRNA levels of BCL9Lwere upregulated in some OS cell lines, especially in MG63 and U2OS cells (*n* = 4). **(H)** 143B and U2OS cells contained the most BCL9L protein compared to the other cell lines (*n* = 3). **(I)** The WT-BCL9L-3′-UTR and MUT-BCL9L-3′-UTR were synthesized. Overexpressed miR-766-3p notably inhibited the luciferase activity of WT-BCL9L-3′-UTR but had no influence on MUT-BCL9L-3′-UTR in 143B and U2OS cells (*n* = 5). **(J)** qRT-PCR indicated that the BCL9L mRNA level was negatively regulated by miR-766-3p (*n* = 4). **(K)** Western blotting supported that miR-766-3p negatively controls the expression level of BCL9L (*n* = 3). Data are presented as the means ± SD. *P < 0.01.

To further detect the potential underlying mechanism of miR-766-3p in OS occurrence and development Potential targets of miR-766-3p were predicted using TargetScan, miRDB, and miRTarBase Tools (Figure 4A). Among all candidate genes, we paid specifically attention to BCL9L because it played essential tumor-promoting role in modulation of carcinogenesis and cancer progression. BCL9L expression in 60 paired OS and peritumor tissues was measured by qRT-PCR and Western blotting, and the expression level of BCL9L was significantly more in tumor than in peritumor tissues (*P* < 0.05, Figures 4B,D). These results were confirmed by immunohistochemistry assays (Figure 4C). As demonstrated in Figure 4E, Kaplan-Meier analysis revealed that the survival rate of OS patients with high expression of BCL9L was significantly lower than that of OS patients with low expression (*P* = 0.0031). Further, BCL9L expression was negatively related with miR-766-3p with an R2 of 0.4751 in OS tissues (Figure 4F). Furthermore, the mRNA level of BCL9L was upregulated in OS cells, particularly in U2OS and 143B cell lines (*P* < 0. 01, Figure 4G). And compared with the other cell lines, U2OS and 143B cells contained the most BCL9L protein (*P* < 0.01, Figure 4H). Besides, Taking the median levels of miR-766-3p and BCL9L mRNA as the critical points, the patients were divided into “high” and “low” subgroups. Table 1 showed that BCL9L was positively related to tumor size, TNM stage, and lung metastasis. Next, we carried out dual-luciferase reporter assays to demonstrate that miR-766-3p could directly targeted BCL9L. It revealed that miR-766-3p overexpression could markedly reduce the luciferase activity of OS cells (Figure 4I). As shown in Figure 4J, quantitative RT-PCR indicated that BCL9L mRNA expression was low in OS cells with miR-766-3p mimics; on the contrary, the high expression of BCL9L was observed in OS cells transfected with miR-766-3p inhibitor. Furthermore, Western blotting assays determined that miR-766-3p negatively regulated the level of BCL9L expression (Figure 4K). In conclusion, these results showed that BCL9Lwas overexpressed in OS cells and a target gene of miR-766-3p.

### BCL9L Promoted EMT, Migration, and Invasion of OS Cells, and Restored the Role of miR-766-3p

**FIGURE 5:**
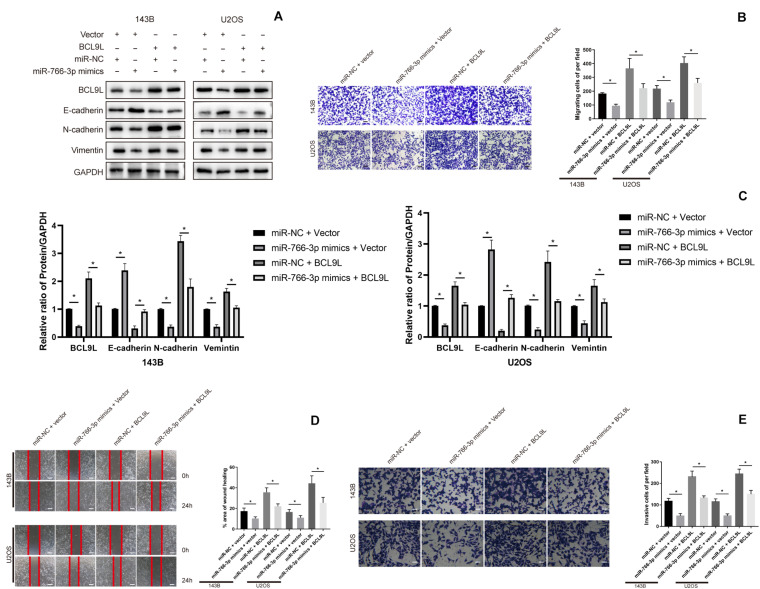
Upregulating BCL9L restored the effects of miR-766-3p mimics on OS cells. **(A,C)** Western blotting showed that OS cells invasion and migration was remedied by overexpressed BCL9L (*n* = 3). **(B,D)** BCL9L could reverse the augmented OS cell invasion and migration caused by miR-766-3p mimics by the transwell assay **(B)** and wound-healing assay **(D)** (*n* = 4). **(E)** These above results were confirmed by cell Transwell invasion assays (*n* = 4). Data are presented as the means ± SD. *P < 0.01.

In order to confirm that miR-766-3p mediates EMT, migration and invasion of OS cells via BCL9L, we did rescue experiments. First, upregulation of miR-766-3p or BCL9L in OS cells was achieved by transfection of miR-766-3p mimics or BCL9L sequences. Then, Western blotting revealed that BCL9L expression in OS cells was positively correlation with the expression of metastasis-related proteins but negatively related with E-cadherin expression. And upregulating miR-766-3p resulted in the opposite result of overexpressing BCL9L. Surprisingly, the influences of overexpressing miR-766-3p were evidently restored with upregulating BCL9L (Figures 5A,C). Transwell migration assays were conducted and the results showed that BCL9L inhibited the protective influences of miR-766-3p mimics on OS patients (*P* < 0.01; Figure 5B); the inhibitory influences of cell migration from miR-766-3p mimics were reduced by BCL9L upregulation in the wound-healing assays (*P* < 0.01; Figure 5D). As shown in Figure 5E, the above results were also verified in Transwell invasion assays. Taken together, BCL9L was confirmed to promote OS cells EMT, migration and invasion, and recover the inhibition of miR-766-3p mimics.

### miR-766-3p Regulated the β-Catenin/TCF-4 Signal Pathway Through BCL9L

**FIGURE 6:**
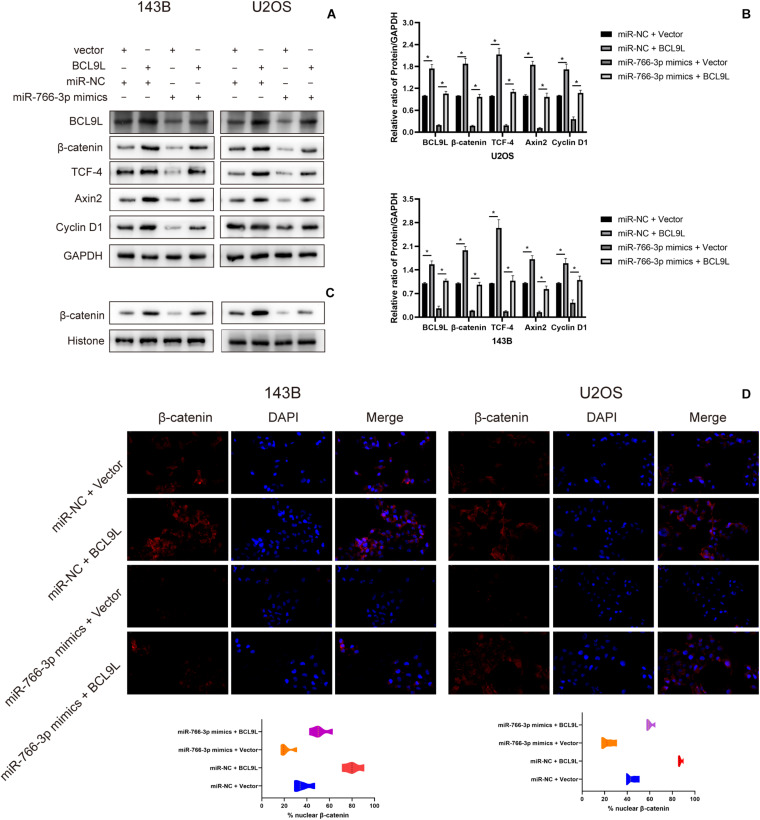
miR-766-3p regulated the β-catenin/TCF-4 signaling pathway via BCL9L. **(A,B)** miR-766-3p mimics decreased the levels of BCL9L together with expression of β-catenin, TCF-4, Cyclin D1, and Axin2, but the suppressing effects were all remedied by overexpressed BCL9L (*n* = 3). **(C)** The levels of β-catenin protein contained in nuclei had a negative correlation with the miR-766-3p expression and were positively correlated with the expression level of BCL9L (*n* = 3). **(D)** miR-766-3p mimics enhanced β-catenin import into the nuclei of OS cells, and BCL9L could abolish this effect (*n* = 4). Data are presented as the means ± SD. *P < 0.01.

To explore the downstream mechanism of miR-766-3p/BCL9L axis regulating cells EMT, migration, and invasion in OS, we performed Western blotting and immunofluorescence analysis. Previous studies had reported that the β-catenin signal pathway was involved in cancer development and was a key process in the occurrence and development of various cancers, including the tumors of skeletal system. So, whether miR-766-3p and BCL9L influenced OS invasion and migration through the β-catenin pathway would to be the focus of our study. Western blotting assays revealed that miR-766-3p mimics in 143B and U2OS cells reduced BCL9L expression, as well as downregulated the levels of β-catenin, TCF-4, Cyclin D1, and Axin2, but the inhibiting effects were all remedied by upregulating BCL9L (*P* < 0.01; Figures 6A,B). Furthermore, the expression of β-catenin protein in the nucleus was negatively related with miR-766-3p, and was positively correlated with BCL9L (Figure 6C). Immunofluorescence assays demonstrated that miR-766-3p mimics induced β-catenin into the nucleus of OS cell and the influence would be recovered by overexpressing BCL9L (*P* < 0.01; Figure 6D). Overall, these results confirmed that the miR-766-3p/BCL9L axis mediated proliferation, EMT and metastasis of OS through the β-catenin/TCF-4 signal pathway.

### miR-766-3p Suppressed the Growth of Xenograft OS Tumors *in vivo*

**FIGURE 7:**
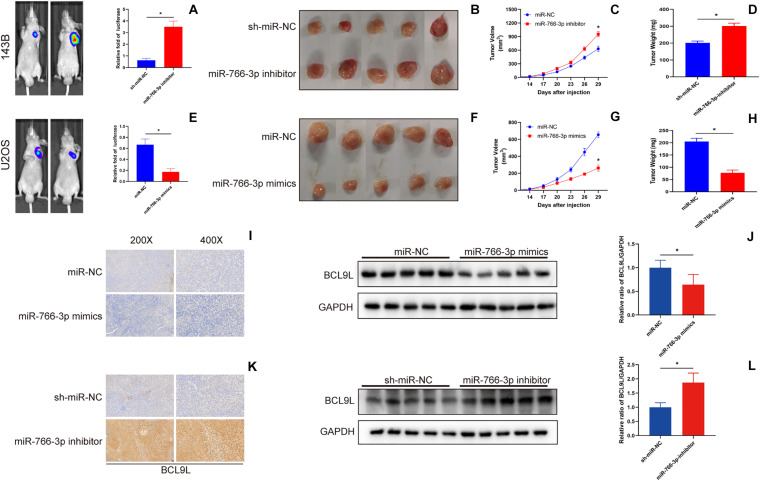
miR-766-3p suppressed xenograft tumor growth and pulmonary metastasis *in vivo*. **(A–D)** Downregulation of miR-766-3p promoted the tumor growth of 143B cells, and tumor volume and average tumor weight were larger and heavier, respectively, than the sh-miR-NC group (*n* = 5). **(E–H)** U2OS cells with high miR-766-3p expression significantly inhibited tumor growth in nude mice. The tumor volume was smaller and the average tumor weight was lighter in the miR-766-3p mimics group (*n* = 5). **(I,J)** The immunohistochemistry assays and western blotting showed that BCL9L expression was reduced in the miR-766-3p mimics group (*n* = 3). **(K,L)** Enhanced BCL9L expression was observed in the miR-766-3p inhibitor group by immunohistochemistry and western blotting assays (*n* = 3). Data are presented as the means ± SD. * *P* < 0.01.

**FIGURE 8:**
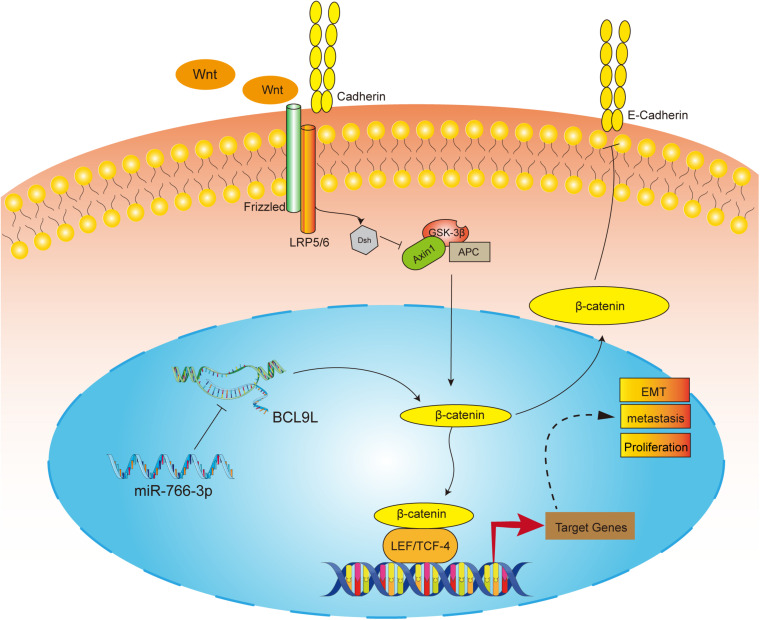
The molecular mechanism underlying the tumor-suppressive effect of miR-766-3p in OS. miR-766-3p targeting BCL9L inhibited proliferation, EMT, and metastasis by down-regulating β-catenin signaling pathway in OS cells.

In order to detect the influence of miR-766-3p *in vivo*, OS cells transfected with miR-766-3p mimic or inhibitor were inoculated into nude mice subcutaneously. The size of the xenograft tumors was observed every 3 days from the 14th day after inoculation, and the mice were sacrificed 5 weeks later. Figures 7A,B showed that knockdown of miR-766-3p accelerated the growth of OS cells (*P* < 0.01), and the tumor volume and weight were larger and heavier respectively (*P* < 0.01, Figure 7C; *P* < 0.01, Figure 7D). On the contrary, high expression of miR-766-3p remarkably suppressed tumor growth compared to controls (*P* < 0.01, Figures 7E,F), and the tumor was smaller and lighter (*P* < 0. 01, Figure 7G; *P* < 0.01, Figure 7H). Then, to examine the expression level of BCL9L in xenografts, immunohistochemistry revealed that the expression level of BCL9L was enhanced by miR-766-3p inhibitor; in contrast, miR-766-3p mimics decreased BCL9L expression (Figures 7I,K). Furthermore, the variation expression of BCL9L in xenografts was confirmed by Western blotting (*P* < 0.01, Figure 7J; *P* < 0.01, Figure 7L). Taken together, miR-766-3p plays a significant role in promoting proliferation of OS cells *in vivo*. As shown in the mechanism diagram, miR-766-3p and BCL9L played a crucial role in the occurrence and development of OS; additionally, miR-766-3p suppressed OS cell proliferation, EMT and metastasis via β-catenin/TCF-4 signal pathway, and directly targeted BCL9L (Figure 8).

## Discussion

As mentioned in the previous studies, osteosarcomas occurring predominantly in children or adolescents are the most frequent primary malignant sarcomas (Aljubran et al., 2009; Lindsey et al., 2017). Although previous researches reveal that non-coding RNA has significant influence on regulating biological processes of various cancers (Lin and Gregory, 2015; Rupaimoole et al., 2016), unfortunately, the influence of non-coding RNA in OS pathogenesis is still unclear. Therefore, to investigate the mechanism of OS development and metastasis and identify novel therapies should be an urgent requirement (Gianferante et al., 2017; Sayles et al., 2019). In the present study, miR-766-3p was lowly expressed in OS tissue specimens and cells, negatively related with OS malignancy, and might be used as a novel treating target for OS.

Emerging research has demonstrated that the abnormal miRNA expression plays key regulatory role in OS progression (Czarnecka et al., 2020; Zhang H. et al., 2020), the potential mechanism of miR-766-3p has not been investigated. miR-766-3p has recently been recognized as a tumor suppressor through the β-catenin pathway in several tumors. miR-766-3p was lower in renal cell carcinoma and hepatocellular carcinoma specimens or cell lines than in normal group (Chen et al., 2017; Liu et al., 2020). Similarly, our findings via real-time quantitative PCR are consistent with those from previous studies. In the current study, it was firstly found that miR-766-3p was downregulated in OS sample tissues and cells, indicating that miR-766-3p might function as an OS suppressor. And a series of experiments were conducted *in vitro*, showing that miR-766-3p inhibited proliferation, migration and invasion of OS cells.

EMT has long been linked to cancer malignant progression, tumor migration and metastasis (Mittal, 2002, 2018). In the process, epithelial tumor cell acquires a mesenchymal phenotype, which is characterized with the loss of adhesion between cells, loss of cell polarity and actin cytoskeleton remodeling. And these central changes enhance migration, invasion of cancer cells and confer resistance to therapy (Lamouille et al., 2014; Dongre and Weinberg, 2019; Jolly et al., 2019). In addition, other characteristics of EMT phenotype are down-regulation of epithelial markers and overexpression of mesenchymal markers (Brabletz et al., 2018). In this study, up-regulation of epithelial marker in OS cells was mediated by an upregulated miR-766-3p level; meanwhile, the levels of mesenchymal markers (Vimentin and N-cadherin) decreased. And miR-766-3p inhibited the ability of OS cell metastasis, indicating that miR-766-3p acted as a suppressing factor in progress of OS. Moreover, we demonstrated that BCL9L is one target gene of miR-766-3p. And it was first demonstrated that miR-766-3p inhibited BCL9L expression at the level of cellular transcription and translation. Then, we found that the luciferase activity of WT-BCL9L-3′-UTR was inhibited in miR-766-3p mimics group. Furthermore, BCL9L was upregulated in OS, knockdown of whose expression could reverse the influences of miR-766-3p.

BCL9L, a protein like B-Cell CLL/Lymphoma 9 (BCL9), is usually regarded as a cofactor of canonical β-catenin signal pathway and induces EMT in mammal (Adachi et al., 2004; Cantù et al., 2017). Previous studies have reported that BCL9L can promote the occurrence and development of choriocarcinoma (Matsuura et al., 2011), breast cancer (Toya et al., 2007), colon cancer (Deka et al., 2010), and pancreatic cancer (Hallas et al., 2016). It was reported that higher expression of BCL9L predict lower survival rates in intestinal tumor patients and could be employed as an independent prognostic biomarker. Additionally, BCL9L was abnormally elevated in 43% of colorectal tumors, and could enhance the transcriptional activity of tumor cell invasion mediated by β-catenin-TCF. Here, our study findings validated that BCL9L was significantly upregulated among OS tissues, relative to the peritumor tissues, indicating that BCL9L had a promotable role in tumorigenesis and progression of OS.

Wnt signaling pathway regulates various processes that are essential for cancer progression, and β-catenin signal pathway plays crucial role in the process (Klaus and Birchmeier, 2008; Nusse and Clevers, 2017). Moreover, β-catenin functions as a transcriptional switch, recruiting transcriptional cofactors including BCL9L, Pygopus, or histone acetyltransferase, and deters the combination between TLE with TCF/LEF (Perugorria et al., 2019; Tong et al., 2020). Considering that, it is interesting to explore whether miR-766-3p suppresses this pathway via BCL9L. The results showed that miR-766-3p mimics reduced the levels of β-catenin, TCF, Cyclin D1, and Axin2, together with the downregulated BCL9L by Western blotting. On the contrary, downregulating miR-766-3p escalated levels of the above proteins. In addition, the application of upregulating BCL9L markedly restored the influences of miR-766-3p mimics on the β-catenin signal pathway. And Immunofluorescence assay further showed that BCL9L could induce more β-catenin importing into the OS cell nucleus, while upregulating miR-766-3p could abolish this effect.

## Conclusion

In summary, we found the inhibitory effect of miR-766-3p on the occurrence and development of osteosarcoma and its potential mechanism via BCL9L and the β-catenin signal pathways. We verified that miR-766-3p suppressed proliferation, EMT and metastasis of OS cells through β-catenin signaling pathway by directly regulating BCL9L *in vitro* and *vivo*. Therefore, it is a believable inference that miR-766-3p/BCL9L signal pathway may provide valuable information for OS targeted therapy, which may be helpful to regulate the proliferation and metastasis of OS, and ultimately improve the patients’ survival rate.

## Data Availability Statement

All datasets generated for this study are included in the article.

## Ethics Statement

The studies involving human participants were reviewed and approved by the Ethics Committee of the First Affiliated Hospital of Nanjing Medical University. Written informed consent to participate in this study was provided by the participants’ legal guardian/next of kin. The animal study was reviewed and approved by the Animal Ethics Committee of Nanjing Medical University. Written informed consent was obtained from the individual(s), and minor(s)’ legal guardian/next of kin, for the publication of any potentially identifiable images or data included in this article.

## Author Contributions

SZ performed the experiments, analyzed the data, and wrote the manuscript. HC, WL, and LF conceived the study and revised the manuscript. ZQ, RK, and QZ performed the experiments and analyzed the data. JL and XC conceived, designed, and supervised the research. All authors contributed to the article and approved the submitted version.

## Conflict of Interest

The authors declare that the research was conducted in the absence of any commercial or financial relationships that could be construed as a potential conflict of interest.

## Footnotes

1
http://targetscan.org
