# Corrigendum: miR-766-3p targeting BCL9L suppressed tumorigenesis, epithelial-mesenchymal transition, and metastasis through the β-catenin signaling pathway in osteosarcoma cells

**DOI:** 10.3389/fcell.2023.1239836

**Published:** 2023-11-15

**Authors:** Sheng Zhang, Hongtao Chen, Wanshun Liu, Le Fang, Zhanyang Qian, Renyi Kong, Qi Zhang, Juming Li, Xiaojian Cao

**Affiliations:** ^1^ Department of Orthopedics, First Affiliated Hospital of Nanjing Medical University, Nanjing, China; ^2^ Department of Respiratory and Critical Care Medicine, First Affiliated Hospital of Nanjing Medical University, Nanjing, China; ^3^ Department of Orthopedics, The Affiliated Zhongda Hospital of Southeast University, Nanjing, China; ^4^ Department of Pain Management, Sir Run Run Hospital, Nanjing Medical University, Nanjing, China

**Keywords:** osteosarcoma, miR-766-3p, BCL9L, β-catenin, EMT, metastasis

In the published article, there was an error in Figure 2 as published. The photograph of U2OS N-cadherin WB strip in Figure 2B, the 143B miR-766-3p sh#2 cell migration and invasion photograph of Figures 2E, G are wrong. The corrected Figure 2 and its caption appear below.

**FIGURE 2:**
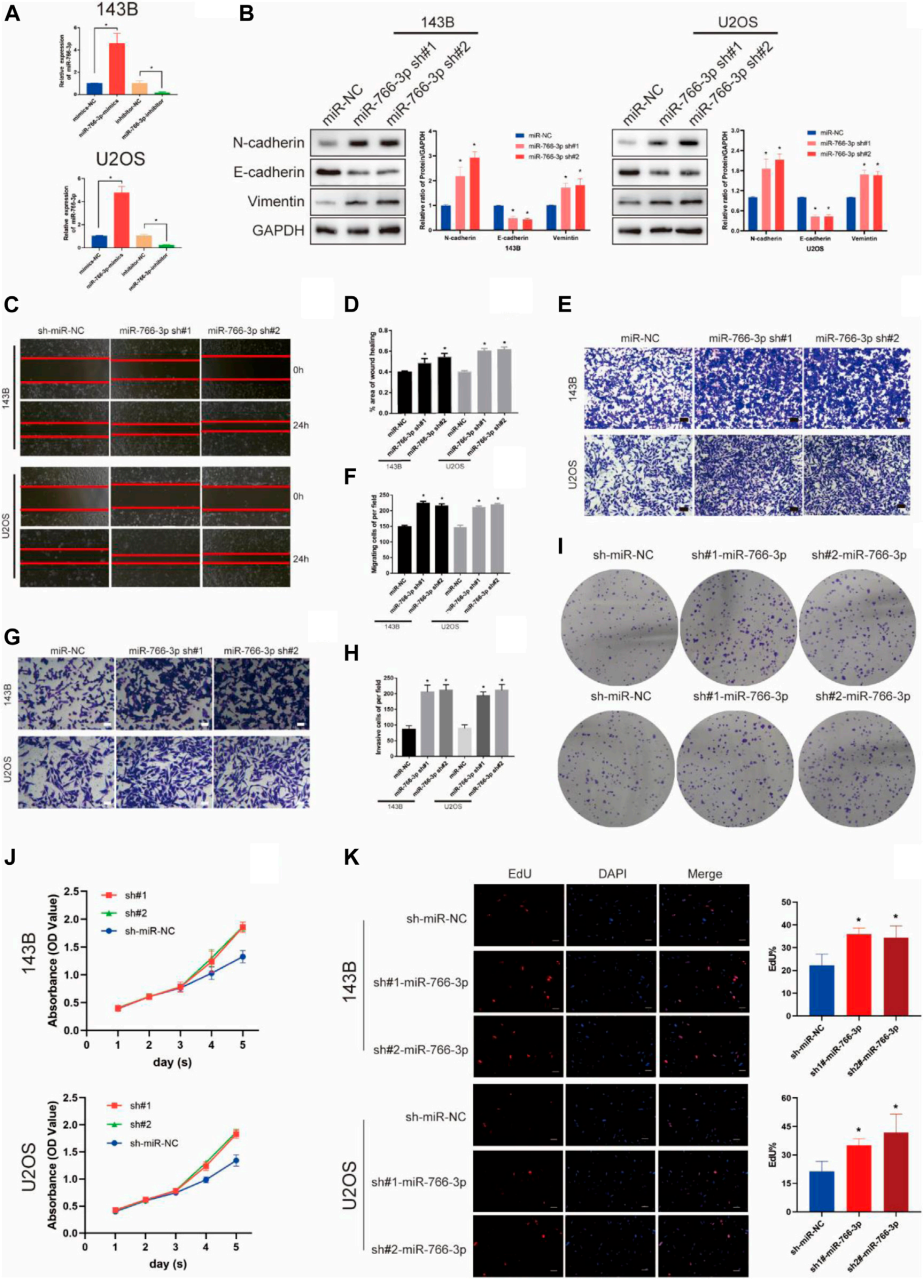
Downregulating miR-766-3p promoted OS cell EMT, migration and invasion *in vitro*. **(A)** miR-766-3p lentiviruses were successfully transfected into 143B and U2OS cell lines (*n* = 3). **(B)** miR-766-3p sh#1 and miR-766-3p sh#2 increased the expression level of metastasis-related proteins in 143B and U2OS (*n* = 3). **(C–F)** The knockdown of miR-766-3p notably promoted the invasion and migration of 143B and U2OS cells (*n* = 4). **(G, H)** The Transwell invasion assays indicated that the knockdown of miR-766-3p significantly increased the invasive ability of OS cells (*n* = 4). **(I–K)** Colony formation, CCK-8 and EdU assays demonstrated that downregulating miR-766-3p promoted the proliferation of OS cells (*n* = 4). Data are presented as the means ± SD. *p < 0.01.

The authors apologize for these errors and state that this does not change the scientific conclusions of the article in any way. The original article has been updated.

